# The Subgingival Microbiome in Patients with Down Syndrome and Periodontitis

**DOI:** 10.3390/jcm9082482

**Published:** 2020-08-02

**Authors:** Lourdes Nóvoa, María del Carmen Sánchez, Juan Blanco, Jacobo Limeres, Maigualida Cuenca, María José Marín, Mariano Sanz, David Herrera, Pedro Diz

**Affiliations:** 1 Medical-Surgical Dentistry Research Group, Health Research Institute of Santiago de Compostela, University of Santiago de Compostela, 15782 Santiago de Compostela, Spain; lourdes.novoa@rai.usc.es (L.N.); jacobo.limeres@usc.es (J.L.); pedro.diz@usc.es (P.D.); 2 Etiology and Therapy of Periodontal Diseases Research Group, University Complutense of Madrid, 28040 Madrid, Spain; mariasb@farm.ucm.es (M.d.C.S.); maiguacu@ucm.es (M.C.); mariajosemarin@gmail.com (M.J.M.); marsan@ucm.es (M.S.); davidher@odon.ucm.es (D.H.)

**Keywords:** Down syndrome, periodontitis, microbiome, microbial community, DNA sequencing

## Abstract

Objective: To describe the subgingival microbiome of individuals with Down syndrome (DS). Methods: We conducted a cross-sectional observational study that obtained bacterial DNA samples from 50 patients with DS, 25 with periodontitis (PDS) and 25 with a healthy periodontal condition (HDS). The samples were analyzed by sequencing the 16S rRNA gene V3–V4 hypervariable region using the MiSeq System. Taxonomic affiliations were assigned using the naïve Bayesian classifier integrated in QIIME2 plugins. We evaluated the difference in bacteria abundance between the sample groups using Wilcoxon and Kruskal–Wallis tests. We evaluated the alpha diversity of the identified species using the Observed, Chao1metric, ACE and Shannon indices and evaluated beta diversity with principal coordinate analysis (registration code: 2018/510). Results: Twenty-one genera and 39 bacterial species showed a significantly different abundance between the study groups. Among the genera, *Porphyromonas*, *Treponema*, *Tannerella* and *Aggregatibacter* were more abundant in the PDS group than in the HDS group, as were the less commonly studied *Filifactor*, *Fretibacterium* and *Desulfobulbus* genera. Among the species, *Porphyromonas* spp. and *Tannerella* spp. were the most abundant in the PDS group; the most abundant species in the HDS group were *Pseudomonas* spp., *Granulicatella* spp. and *Gemella* spp. Conclusion: Well-recognized periodontal pathogens and newly proposed pathogenic taxa were associated with periodontitis in patients with DS.

## 1. Introduction

Down syndrome (DS) is a genetic disease caused by autosomal genetic disorders, mainly the presence of an extra chromosome in pair 21 [1]. Patients with DS have a characteristic phenotype associated with intellectual disability [2] and often have immunological disorders and systemic conditions, such as congenital heart disease, thyroid dysfunction and oral diseases [3].

Based on the current classification of periodontal diseases [4], periodontitis in individuals with DS would be classified as periodontitis as a manifestation of systemic disease, given that the systemic condition affects the course of periodontitis, resulting in an early onset of severe periodontitis [5]. In these patients, it has been suggested that a particular subgingival microbiota [6,7], together with a defective host-response inherent to DS [8], could induce dysbiosis, thus favoring the early onset of periodontitis [9].

There is scarce scientific evidence of a specific subgingival microbiota in patients with DS and periodontitis, and the few available studies have reported conflicting results. A number of microbiological studies on patients with DS have reported a specific microbiological pattern, with *Tannerella forsythia* the predominant bacterial species, followed by *Treponema denticola* and *Porphyromonas gingivalis*, and with a prevalence significantly higher in patients with periodontitis, compared with patients with healthy periodontium [7]. In other studies, however, the microbiota showed no specific pattern [6]. These differences could be due to the differing technologies employed to identify the bacteria: DNA probes and checkerboard DNA–DNA hybridization [6] and polymerase chain reaction (PCR) [7]. Although these methods are very specific, they are not sensitive enough to detect low numbers of bacteria and cannot identify nonspecific bacteria.

With the use of the reference gene, massive sequencing techniques and bioinformatics tools for treating the data, we have been able to obtain unprecedented information on the human microbiome, in terms of the taxonomy and function of microorganisms [10]. The aim of this study is to use next-generation sequencing (NGS) to study the subgingival microbiota of patients with DS to determine whether those patients with periodontitis have a differential pattern when compared with patients with DS but no periodontitis.

## 2. Materials and Methods

### 2.1. Study Design and Population

We designed a cross-sectional observational study for a White Spanish population with DS. The study was conducted in accordance with the Declaration of Helsinki of the World Medical Association (2008) and approved by the Research Ethics Committee of Santiago-Lugo, Spain (registration code: 2018/510). All patients (and their legal guardians, where appropriate) signed a consent form.

Between February 2019 and June 2019, all individuals with DS who regularly attended educational or occupational therapy centers in Galicia, Spain, were consecutively selected (Figure S1). The exclusion criteria were (1) age younger than 18 years; (2) coexistence of systemic diseases that could affect periodontal health; (3) presence of harmful habits (e.g., smoking); and (4) having taken systemic antibiotics and/or antimicrobial mouth rinses within the previous 3 months.

### 2.2. Periodontal Examination

After the recruitment, the patients or their guardians provided information on the patients’ age, sex, presence of comorbidities and use of current medications. The patients then underwent an intraoral and periodontal clinical examination, during which the following periodontal variables were recorded for the 4 sites at the 6 reference teeth [11] or, when absent, at the adjacent tooth: plaque index [12], probing depth (PD), bleeding on probing (BOP) [13], gingival recession and clinical attachment level (CAL). These variables were recorded by a trained and calibrated clinical investigator (NL). Calibration was performed over several sessions of repeated measurements, until the clinical investigator obtained intra-class correlation coefficients > 0.75.

The case definition of a patient with a healthy periodontal condition was based on a PD < 4 mm and BOP detected at no more than 2 sites (less than 10% of the total) [14]. A case of periodontitis was defined when an interdental CAL was detected at 2 or more non-adjacent teeth or when a buccal or oral CAL ≥ 3 mm with pocketing > 3 mm was detected at 2 or more teeth [15]. Applying these clinical criteria, we selected a convenience sample consisting of 50 individuals: 25 patients with DS with periodontitis (PDS) and 25 patients with DS with a healthy periodontal condition (HDS).

### 2.3. Microbiological Examination

#### 2.3.1. Subgingival Biofilm Sample Collection

Samples were taken from 4 sites, one in each quadrant, selecting those with the deepest PD and BOP [16]. The sample collection procedure consisted of isolating the selected area with cotton rolls, removing the supragingival plaque or calculus. Once each site had been dried with cotton pellets, two medium-sized sterile paper tips (Maillefer, Ballaigues, Switzerland) were inserted in each site for 10 s, as subgingivally as possible [16]. All paper tips were then pooled in a vial containing 1.5 mm of reduced transport fluid [17] and sent to the Microbiology Laboratory of the Faculty of Dentistry, Complutense University, Madrid to be processed within 24 h.

#### 2.3.2. Extraction of Total Genomic DNA

Total DNA was extracted from subgingival samples using a commercial kit (MoIYsis Complete 5. Molzym Gmbh & Co. KG. Bremen, Germany) following the manufacturer’s instructions (the protocol for bacterial DNA extraction was followed from step 6, skipping the preliminary steps). The extracted DNA was eluted in 100 μL of sterile water (Roche Diagnostic GmbH; Mannheim, Germany) and frozen at −20 °C for further analysis. Two negative control blanks, which included no sample, were subjected to all steps of the DNA extraction and purification procedure described above.

#### 2.3.3. 16S rRNA Gene Sequencing with Illumina^®^ Sequencing

Bacterial DNA from the 50 participants was analyzed by sequencing the 16S rRNA gene V3–V4 hypervariable region using the MiSeq System (Illumina, San Diego, CA, USA). The selection of the V3–V4 region was based on the fact that it provides a more accurate characterization of the microbiome samples than V1–V2 [18] and V1–V3 [19]. The 16S rDNA gene amplicons were obtained following the 16S rDNA gene Metagenomic Sequencing Library Preparation Illumina protocol (Cod. 15044223 Rev. A: Illumina). Illumina adapter overhang nucleotide sequences were added to the gene-specific sequences. The primers were selected from Klindworth et al. [20] [Forward Primer = 5′ TCG TCG GCA GCG TCA GAT GTG TAT AAG AGA CAG CCT ACG GGN GGC WGC AG; Reverse Primer = 5′ GTC TCG TGG GCT CGG AGA TGT GTA TAA GAG ACA GGA CTA CHV GGG TAT CTA ATCC]. Microbial genomic DNA (5 ng/μL in 10 mMTris pH 8.5) was employed to start the protocol. After 16S rDNA gene amplification, the multiplexing step was performed using Nextera XT DNA Library Preparation Kit (FC-131-1096; Illumina). We ran 1 μL of the PCR product in a Bioanalyzer DNA 1000 chip to verify the size. The libraries were then sequenced using a 2 × 300 pb paired-end run (MiSeq Reagent kit v3 (MS-102-3001); Illumina) on a MiSeq Sequencer according to the manufacturer’s instructions (Illumina). Quality assessment was performed using the prinseq-lite program [21]. Data were obtained using an ad hoc pipeline written in the RStatistics environment [22], making use of several open source libraries. Data were grouped and stratified according to the metadata file provided by the user.

The analysis was performed at the Foundation for the Promotion of Health and Biomedical Research of the Valencia Region, Valencia, Spain.

#### 2.3.4. Taxonomic Assignment

We analyzed the sequence data obtained from MiSeq using the QIIME2 pipeline [23]. In brief, denoising, paired-ends joining and chimera depletion was performed starting from paired-ends data using the DADA2 pipeline [24]. Taxonomic affiliations were assigned using the naïve Bayesian classifier integrated in the QIIME2 plugins, using a 97% similarity threshold. The database used for this taxonomic assignation was SILVA_release_132 [25].

### 2.4. Statistical and Bioinformatics Analysis

A sample size calculation was not possible because no previous information was available on the microbiomes of the patients with DS. We selected a convenience sample of 25 patients with PDS, similar to the 30 patients with periodontitis and DS included by Martinez-Martinez et al. [7].

A subject-level analysis was performed for each demographic and clinical variable in the study. The data are expressed as mean and standard deviation (SD), prevalence and proportions (%). After assessing the normality of the variables’ distribution (applying the Kolmogorov–Smirnov test), we compared the results obtained between the PDS and HDS groups using Student’s *t* test for the quantitative variables and the chi-squared test or Fisher’s exact test for the categorical variables. The statistical significance level was set at *p* < 0.05. We employed the IBM^®^SPSS Statistics 21.0 statistics package (IBM Corporation, Armonk, NY, USA) for the data analysis.

In terms of the diversity and significance analysis, we subjected the sample data obtained in the biological observation matrix format to statistical analysis using the R statistics environment [22]. We analyzed the samples’ sequencing depth using the Shannon index. We also estimated the main bacteria and taxonomic composition of the microbiota in each sample. The differences in bacteria abundance between the sample groups were evaluated using Wilcoxon rank-sum and Kruskal–Wallis tests among various metadata factors. Applying the phyloseq R package, we employed 4 non-phylogeny-based metrics, namely the Observed species, Chao1metric, abundance-based coverage estimators (ACE) and Shannon index to evaluate alpha diversity, which represented the amount of diversity contained within communities. We applied a principal coordinate analysis (PCoA) with weighted UniFrac distances to evaluate beta diversity, which represented the amount of diversity shared among communities.

We followed the Strengthening the Reporting of Observational Studies in Epidemiology (STROBE) checklist when reporting the results of the present study (Scheme S1).

## 3. Results

### 3.1. Patients Sample

The original sample included 168 patients with DS with an age range of 18–45 years. In the selected sample, the mean age was not statistically significant between the study groups (*p* = 0.063). The sex distribution was similar, and there were no differences in the prevalence of cardiovascular disease and other systemic conditions. However, thyroid disease showed a greater presence in the PDS group (*p* = 0.008) (Table 1).

### 3.2. Clinical Outcome Variables

The periodontal clinical outcomes were significantly different (*p* < 0.001) between the PDS and HDS groups for PD, CAL, BOP and the plaque index (Table 2).

### 3.3. Overall Microbial Sequencing Results

Following the DNA extraction and bar-coded PCR amplification, the samples were sequenced. After quality filtering, denoising, paired-ends joining and chimera filtering, 50 samples (25 PDS, 25 HDS) were included in the analysis. A total of 5,623,126 reads (median/sample, 114,757; range, 85,827–142,134) were assigned into operational taxonomic units (OTUs). The sequencing depth was similar between the two groups: 2,226,567 reads for HDS and 2,331,571 reads for PDS. The OTUs were classified into 13 phyla: *Firmicutes* (37.17%), *Bacteroidetes* (19.04%), *Proteobacteria* (18.20%), *Fusobacteria* (15.04%), *Actinobacteria* (3.70%), *Spirochaetes* (2.79%), *Epsilonbacteraeota* (2.10%), *Patescibacteria* (1.36%), *Synergistetes* (0.51%), *Chloroflexi* (0.04%), *Tenericutes* (0.03%), *Cyanobacteria* (0.02%) and *Elusimicrobia* (0.01%). These OTUS were further classified into 20 classes, 41 orders, 75 families, 158 genera and 403 species. The diversity is summarized in Figure S2 and was generated using the Krona hierarchical browser [26].

### 3.4. Taxonomic Composition of Periodontitis and Healthy Periodontal Patients

Table 3 summarizes the dominant bacteria in the subgingival microbiome of the patients with DS. The rarefaction analysis showed that the overall diversity was covered by the obtained sequences, indicating that the microbial composition for each periodontal status was well represented by the sequencing depth (Figure S3A), showing rarefaction curves at the phylum, genus and species levels. In terms of community diversity, the PDS and HDS groups presented distinct microbial profiles at all taxonomic levels, although there were no statistically significant differences in overall diversity (Figure S3B) at the phylum, genus and species levels. In terms of the relative abundance in the groups, there were some significant differences (*p* ≤ 0.05) at various taxonomic levels, specifically in 3 phyla, 5 classes, 4 orders, 2 families, 21 genera and 39 species (Table S1).

In the HDS group, the five most abundant phyla were *Firmicutes* (38.00%), *Proteobacteria* (28.23%), *Bacteroidetes* (14.56%), *Fusobacteria* (9.42%) and *Actinobacteria* (4.11%), which represented more than 94% of the total sequences (Table 3). In the PDS group, four of the aforementioned phyla also constituted the majority of the microbiome: *Firmicutes* (44.04%), *Bacteroidetes* (20.23%), *Proteobacteria* (12.04%) and *Fusobacteria* (11.09%), although the abundance of *Spirochaetes* (4.11%) was higher. The five phyla represented approximately 90% of all sequences (Table 3). Three of the 13 identified phyla showed a significantly different abundance between the sample groups: *Chloroflexi* (*p* = 0.0114) and Synergistetes (*p* = 0.0370), which were more abundant in the PDS group, and *Proteobacteria* (*p* = 0.0407), which was more abundant in the HDS group (Table S1). 

At the genus level, the five most abundant genera in the HDS group were *Streptococcus* (19.55%), *Fusobacterium* (6.72%), *Neisseria* (6.15%), *Pseudomonas* (5.73%) and *Veillonella* (4.56%) (Table 3). In contrast, *Streptococcus* (23.55%), *Fusobacterium* (7.48%), *Neisseria* (5.93%), *Porphyromonas* (4.59%) and *Treponema* (4.10%) were the most abundant genera in the PDS group (Table 3). The PDS group harbored higher levels of recently described new putative pathogens, such as species of the genera *Filifactor*, *Fretibacterium* and *Desulfobulbus* [27]. In total, 21 genera were identified with a significantly different abundance between the groups, including *Paludibacteraceae F0058* (*p* < 0.001), *Tannerella* (*p* = 0.029) and *Fretibacterium* (*p* = 0.0371) with a significant abundance in the PDS group compared with the HDS group, as well as *Pseudomonas* (*p* = 0.0028), which were more abundant in the HDS group (Table S1).

Table 3 shows the dominant species in both groups. *Streptococcus* spp. was the most abundant species in both the HDS and PDS groups (17.83% and 21.5%, respectively), followed by *Fusobacterium* spp. (6.72% and 7.35%, respectively). The most abundant species in the HDS group included those of the *Pseudomonas*, *Granulicatella* and *Gemella* genera. In the PDS group, in contrast, the most abundant species were those of the *Porphyromonas* and *Tannerella* genera. Table S1 shows the 39 bacterial species with significant differences between the HDS and PDS groups. 

As shown in Figure S3B, the average richness measured by the Observed, Chao1 and ACE indices was higher in the samples from the PDS group than in samples from the HDS group, although without statistical significance. The ACE and Shannon diversity indices had an identical trend. Thus, the relative abundance of each bacterium was more balanced in the samples from the HDS group than in samples from the PDS group, and there were more bacteria with low relative abundance in the samples from the PDS group. The Simpson index confirms this fact, demonstrating greater uniformity at all taxonomic levels in the HDS group, although without significant differences.

To evaluate the similarity of the microbial community structure among the samples and thereby explore the relationships between the bacterial communities in the PDS and HDS groups, we performed a PCoA. The results showed a distinction between the microbial compositions of the two groups at all taxonomic levels, given that the PDS and HDS groups could not be grouped into one cluster (Figure S4). The HDS group appeared more clustered, unlike the PDS group, which appeared more dispersed. The clusters showed that dispersion was produced by 20% of the periodontitis samples, which presented a dissimilar bacterial community among all samples.

## 4. Discussion

The present study aimed to describe (through the use of NGS) the subgingival microbiome of patients with DS and periodontitis and compare it with the microbiome of patients with DS without periodontitis. The results have shown significant differences between the oral microbiome of the HDS and PDS groups at various taxonomical levels. 

The PDS group harbored higher levels of well-recognized periodontal pathogens, such as *Tannerella*, *Treponema*, *Porphyromonas* and *Aggregatibacter* (*p* < 0.05), as well as recently described new putative pathogens, such as *Peptostreptococcus*, *Filifactor*, *Fretibacterium* and *Desulfobulbus* [25], all of which demonstrated greater relative abundance at the genus level in the PDS group. In contrast, a set of bacterial species, including *Veillonella*, *Neisseria*, *Gemella* and *Granulicatella* were significantly frequent in the samples from the HDS group. These results agree with those of studies on the subgingival microbiota and microbiome in the general population [28,29,30,31,32]. However, there are some discrepancies, mainly in certain species considered compatible with periodontal health such as those from the *Streptococcus* and *Selenomonas* genera that were more abundant in the samples from the PDS group than from the HDS group. We found higher levels of alpha diversity in the PDS group when compared with the HDS group, although these differences were not statistically significant. This diversity might be associated with the patients’ reduced immune competence or with a richer environment in nutrients as a result of the onset of periodontitis, which is in line with previous studies that reported higher bacterial community diversity in different “stages” of periodontitis, when compared with individuals with a healthy periodontal condition [33,34,35].

Although various studies have characterized the subgingival microbiome in patients with and without periodontitis using metagenomic approaches [36,37], the present study is, to the best of our knowledge, the first to evaluate the subgingival microbiome of patients with DS using NGS technology. Our findings corroborate those of previous studies that reported higher mean counts of all “red complex” species and lower counts of *Actinomyces* in patients with periodontitis using checkerboard DNA–DNA hybridization [6], as well as a higher prevalence of *Tannerella*, *Treponema*, *Porphyromonas* and *Aggregatibacter* genera in patients with periodontitis (using PCR) [7]. It stands to reason that the analysis performed using NGS in the present study not only corroborates the results using other techniques but also makes the techniques more comprehensive because the use of NGS techniques results in high throughputs, enabling the processing of thousands or even millions of sequences, which can result in the inaccurate identification of microbial taxa, including uncultivable organisms and those present in small numbers.

The results from this study, however, also present certain limitations. At the microbiological level, certain taxa cannot be distinguished with species-level taxonomic resolution when the 16S rRNA sequencing employed the QIIME pipeline for the downstream taxonomic classification, which might result in some unclassified OTUS such as *Porphyromonas*_Unidentified at the species level [38,39,40,41]. To solve this limitation, other authors have analyzed the reads generated by MiSeq with HOMINGS, an in silico probe-based platform [42].

At the clinical level, the periodontal diagnosis was based on the clinical assessment restricted to the Ramfjord teeth, which were chosen to facilitate the examination of the special patient population included in the study and due to the well-known evidence that partial-mouth recording systems might present adequate accuracy. Differences at baseline (although not statistically significant) should also be discussed as a limitation because the PDS group was slightly older, while the HDS group more frequently had thyroid diseases and were taking drugs for the condition. Finally, a larger sample size would have been desirable; however, there were no previous studies for a proper sample size calculation. We therefore selected a convenience sample [7]. 

## 5. Conclusions

Considering the abovementioned limitations of this cross-sectional study, the use of 16S rRNA-based NGS analysis of subgingival samples from patients with Down syndrome suggests a quite distinct subgingival microbiome in periodontitis versus that of periodontally healthy controls. The PDS group showed a more complex bacterial composition with a higher number of pathogens but not at the expense of a depletion of host-compatible species. Well-recognized periodontal pathogens (including *Porphyromonas*, *Treponema*, *Tannerella* and *Aggregatibacter*) as well as newly proposed pathogenic taxa (*Peptostreptococcus*, *Filifactor*, *Fretibacterium* and *Desulfobulbus*) were associated with periodontitis in the patients with Down syndrome.

## Figures and Tables

**Table 1:** Patient sample characteristics (age, sex and systemic conditions) for the entire sample and for those with Down syndrome and periodontitis or a healthy periodontium.

		Full Sample	HDS	PDS	*p* *
Age, years	mean (SD)	26.8 (6.56)	25.1 (6.18)	28.6 (6.61)	0.063
Sex	Male, *n* (%)	29 (58)	14 (56)	15 (60)	0.774
	Female, *n* (%)	21 (42)	11 (44)	10 (40)	0.774
Systemic disease	all conditions, *n* (%)	21 (42)	11 (44)	10 (40)	0.774
	heart disease, *n* (%)	6 (12)	1 (4)	5 (20)	0.189
	thyroid disease, *n* (%)	12 (24)	10 (40)	2 (8)	0.008
	thyroid medication, *n* (%)	12 (24)	10 (40)	2 (8)	0.008
	other conditions, *n* (%)	6 (12)	3 (12)	3 (12)	1.000

* Student *t*-test for age; chi-squared test/Fisher’s exact test for categorical variables. Abbreviations: HSD, Down syndrome with healthy periodontium; PSD, Down syndrome with periodontitis; SD, standard deviation.

**Table 2:** Periodontal clinical outcome variables, for the whole sample and for the patients with Down syndrome and periodontitis or a healthy periodontium.

	Whole Sample		HDS		PDS		*p* *
	Mean	SD	Mean	SD	Mean	SD	
PD	2.39	0.47	2.07	0.11	2.72	0.47	<0.0001
CAL	2.45	0.59	2.08	0.13	2.81	0.65	<0.0001
REC	0.08	0.25	0.02	0.06	0.14	0.35	0.0985
BOP	19.9	17.8	7.7	7.7	32.2	16.7	<0.0001
PlI	56.5	31.5	41.8	30.3	71.2	25.8	0.0006

Abbreviations: BOP, bleeding on probing; HSD, Down syndrome with healthy periodontium; CAL, clinical attachment level; PlI, plaque index PD, probing depth; PSD, Down syndrome with periodontitis; REC, gingival recession; SD, standard deviation. * Student *t* test.

**Table 3:** Dominant operational taxonomic units and their proportions (in percentage) in the patients with Down syndrome and periodontitis or a healthy periodontal condition, at all taxonomic levels.

**Phylum**				**Class**			
**HDS**		**PDS**		**HDS**		**PDS**	
*Firmicutes*	38.00	*Firmicutes*	44.04	*Gammaproteobacteria*	28.16	*Bacilli*	29.27
*Proteobacteria*	28.23	*Bacteroidetes*	20.23	*Bacilli*	26.63	*Bacteroidia*	20.23
*Bacteroidetes*	14.56	*Proteobacteria*	12.04	*Bacteroidia*	14.56	*Gammaproteobacteria*	11.83
*Fusobacteria*	9.42	*Fusobacteria*	11.09	*Fusobacteriia*	9.42	*Fusobacteriia*	11.09
*Actinobacteria*	4.11	*Spirochaetes*	4.10	*Negativicutes*	7.67	*Negativicutes*	8.66
*Epsilonbacteraeota*	2.58	*Actinobacteria*	3.71	*Actinobacteria*	4.04	*Spirochaetia*	4.10
*Spirochaetes*	1.78	*Epsilonbacteraeota*	2.49	*Clostridia*	3.65	*Clostridia*	6.02
*Patescibacteria*	1.17	*Patescibacteria*	1.74	*Campylobacteria*	2.58	*Actinobacteria*	3.42
*Synergistetes*	0.11	*Synergistetes*	0.39	*Spirochaetia*	1.78	*Campylobacteria*	2.49
*Cyanobacteria*	0.03	*Chloroflexi*	0.08	*Gracilibacteria*	0.16	*Coriobacteriia*	0.30
**Order**				**Family**			
**HDS**		**PDS**		**HDS**		**PDS**	
*Lactobacillales*	23.63	*Lactobacillales*	26.69	*Streptococcaceae*	19.56	*Streptococcaceae*	23.55
*Betaproteobacteriales*	10.60	*Bacteroidales*	16.02	*Neisseriaceae*	7.67	*Veillonellaceae*	8.66
*Fusobacteriales*	9.42	*Fusobacteriales*	11.09	*Veillonellaceae*	7.06	*Fusobacteriaceae*	7.48
*Bacteroidales*	9.40	*Selenomonadales*	8.66	*Fusobacteriaceae*	6.72	*Neisseriaceae*	7.23
*Selenomonadales*	7.67	*Betaproteobacteriales*	7.71	*Prevotellaceae*	5.75	*Prevotellaceae*	6.13
*Pseudomonadales*	5.73	*Clostridiales*	6.02	*Pseudomonadaceae*	5.73	*Porphyromonadaceae*	4.59
*Pasteurellales*	5.35	*Spirochaetales*	4.10	*Pasteurellaceae*	5.35	*Spirochaetaceae*	4.10
*Flavobacteriales*	4.87	*Pasteurellales*	3.5	*Carnobacteriaceae*	3.77	*Leptotrichiaceae*	3.61
*Clostridiales*	3.65	*Flavobacteriales*	3.37	*Enterobacteriaceae*	3.47	*Pasteurellaceae*	3.50
*Enterobacteriales*	3.47	*Bacillales*	2.57	*Flavobacteriaceae*	3.32	*Flavobacteriaceae*	3.16
**Genus**				**Species**			
**HDS**		**PDS**		**HDS**		**PDS**	
*Streptococcus*	19.56	*Streptococcus*	23.55	*Streptococcus u. spp.a*	17.83	*Streptococcus u. spp.*	21.48
*Fusobacterium*	6.72	*Fusobacterium*	7.48	*Fusobacterium u. spp.*	6.72	*Fusobacterium u. spp.*	7.35
*Neisseria*	6.15	*Neisseria*	5.93	*Pseudomonas u. spp.*	5.73	*Neisseria u. spp.*	4.73
*Pseudomonas*	5.73	*Porphyromonas*	4.59	*Granulicatella u. spp.*	3.63	*Veillonella u. spp.*	4.11
*Veillonella*	4.56	*Treponema 2*	4.10	*Neisseria u.b.*	3.28	*Paludibacteraceae F0058 u. spp.*	3.05
*Granulicatella*	3.77	*Veillonella*	4.10	*Haemophilus u. spp.*	2.59	*Granulicatella u. spp.*	2.86
*Haemophilus*	3.33	*Leptotrichia*	3.61	*Delftia u. spp.*	2.52	*Porphyromonas u. spp.*	2.23
*Capnocytophaga*	3.18	*Capnocytophaga*	3.16	*Neisseria u. spp.*	2.44	*Gemella u. spp.*	1.89
*Gemella*	2.99	*Gemella*	2.57	*Stenotrophomonas u. spp.*	2.28	*Tannerella u. spp.*	1.56
*Leptotrichia*	2.71	*Granulicatella*	2.91	*Gemella u. spp.*	1.79	*Porphyromonas u. spp.*	1.43

Abbreviations: HSD, Down syndrome with healthy periodontium; PSD, Down syndrome with periodontitis.

## Supplementary Materials

The following are available online at https://www.mdpi.com/2077-0383/9/8/2482/s1. The following files will be loaded as supplementary material in the submission procedure: Scheme S1. STROBE Statement—Checklist of items that should be included in reports of cross-sectional studies. Figure S1: Flow diagram for the selection of individuals with Down syndrome included in this study with periodontitis (PDS) and healthy periodontium (HDS), Figure S2: Summary of the overall diversity in the patients with Down syndrome with periodontitis (A) and healthy periodontium (B), at all taxonomic levels, Figure S3: (A) Rarefaction curves and (B) principal coordinate analysis of the microbial composition of samples according to disease status (periodontitis [case] and healthy periodontal condition [control]), based on phylum level, genus and species data, Figure S4: Beta diversity analysis. Plots were generated using weighted UniFrac distances. Circles in green and orange represent different periodontal bacterial community clusters from periodontitis (case) and healthy periodontal condition (control), respectively, at all taxonomic levels, Table S1: Differences in microbial relative abundance (in percentages) evaluated using the Wilcoxon and Kruskal–Wallis tests between samples from patients with Down syndrome and periodontitis or a healthy periodontal condition at the phylum, genus and species levels. Only taxa that were significantly different between the two groups (*p* ≤ 0.05) are presented.

## References

[1] Lejeune J., Gautier M., Turpin R. (1959). Etude des chromosomes somatiques de neuf enfants mongoliens. [Study of somatic chromosomes from 9 mongoloid children]. C. R. Hebd. Seances Acad. Sci..

[2] Abanto J., Ciamponi A.L., Francischini E., Murakami C., De Rezende N.P., Gallottini M. (2011). Medical problems and oral care of patients with Down syndrome: A literature review. Spec. Care. Dentist..

[3] Bloemers B.L., van Bleek G.M., Kimpen J.L., Bont L. (2010). Distinct abnormalities in the innate immune system of children with Down syndrome. J. Pediatr..

[4] Caton J.G., Armitage G., Berglundh T., Chapple I.L.C., Jepsen S., Kornman K.S., Mealey B.L., Papapanou P.N., Sanz M., Tonetti M.S. (2018). A new classification scheme for periodontal and peri-implant diseases and conditions—Introduction and key changes from the 1999 classification. J. Clin. Periodontol..

[5] Jepsen S., Caton J.G., Albandar J.M., Bissada N.F., Bouchard P., Cortellini P., Demirel K., de Sanctis M., Carlo Ercoli C., Fan J. (2018). Periodontal manifestations of systemic diseases and developmental and acquired conditions: Consensus report of workgroup 3 of the 2017 World Workshop on the Classification of Periodontal and Peri-Implant Diseases and Conditions. J. Clin. Periodontol..

[6] Khocht A., Yaskell T., Janal M., Turner B.F., Rams T.E., Haffajee A.D., Socransky S.S. (2012). Subgingival microbiota in adult Down syndrome periodontitis. J. Periodontal. Res..

[7] Martinez-Martinez R.E., Loyola-Rodriguez J.P., Bonilla-Garro S.E., Patiño-Marin N., Haubek D., Amano A., Poulsen K. (2013). Characterization of periodontal biofilm in Down syndrome patients: A comparative study. J. Clin. Pediatr. Dent..

[8] Khocht A., Russell B., Cannon J.G., Turner B., Janal M. (2014). Oxidative burst intensity of peripheral phagocytic cells and periodontitis in Down syndrome. J. Periodontal. Res..

[9] Hajishengallis G., Lamont R.J. (2012). Beyond the red complex and into more complexity: The polymicrobial synergy and dysbiosis (PSD) model of periodontal disease etiology. Mol. Oral. Microbiol..

[10] Xu P., Gunsolley J. (2014). Applications of metagenomics in understanding oral health and disease. Virulence.

[11] Ramfjord S.P. (1959). Indices for Prevalence and Incidence of Periodontal Disease. J. Periodontol..

[12] O’Leary T.J., Drake R.B., Naylor J.E. (1972). The plaque control record. J. Periodontol..

[13] Ainamo J., Barmes D., Beagrie G., Cutress T., Martin J., Sardo-Infirri J. (1982). Development of the World Health Organization (WHO) community periodontal index of treatment needs (CPITN). Int. Dent. J..

[14] Chapple I.L.C., Mealey B.L., Van Dyke T.E., Bartold P.M., Dommisch H., Eickholz P., Geisinger M.L., Genco R.J., Glogauer M., Goldstein M. (2018). Periodontal health and gingival diseases and conditions on an intact and a reduced periodontium: Consensus report of workgroup 1 of the 2017 World Workshop on the Classification of Periodontal and Peri-Implant Diseases and Conditions. J. Clin. Periodontol..

[15] Tonetti M.S., Greenwell H., Kornman K.S. (2018). Staging and grading of periodontitis: Framework and proposal of a new classification and case definition. J. Clin. Periodontol..

[16] Mombelli A., McNabb H., Lang N.P. (1991). Black-pigmenting gram-negative bacteria in periodontal disease. II. Screening strategies for detection of P. gingivalis. J. Periodontal. Res..

[17] Syed S.A., Loesche W.J. (1972). Survival of human dental plaque flora in various transport media. Appl. Microbiol..

[18] Teng F., Darveekaran Nair S.S., Zhu P., Li S., Huang S., Li X., Xu J., Yang F. (2018). Impact of DNA extraction method and targeted 16S-rRNA hypervariable region on oral microbiota profiling. Sci. Rep..

[19] Fouhy F., Clooney A.G., Stanton C., Claesson M.J., Cotter P.D. (2016). 16S rRNA gene sequencing of mock microbial populations—Impact of DNA extraction method, primer choice and sequencing platform. BMC Microbiol..

[20] Klindworth A., Pruesse E., Schweer T., Peplies J., Quast C., Horn M., Glöckner F.O. (2013). Evaluation of general 16S ribosomal RNA gene PCR primers for classical and next-generation sequencing-based diversity studies. Nucleic Acids. Res..

[21] Schmieder R., Edwards R. (2011). Quality control and preprocessing of metagenomic datasets. Bioinformatics.

[22] R Core Team (2012). R: A Language and Environment for Statistical Computing. R: The R Project for Statistical Computing.

[23] Caporaso J.G., Kuczynski J., Stombaugh J., Bittinger K., Bushman F.D., Costello E.K., Fierer N., Peña A.G., Goodrich J.K., Gordon J.I. (2010). QIIME allows analysis of high-throughput community sequencing data. Nat. Methods.

[24] Callahan B.J., McMurdie P.J., Rosen M.J., Han A.W., Johnson A.J., Holmes S.P. (2016). DADA2: High-resolution sample inference from Illumina amplicon data. Nat. Methods.

[25] Quast C., Pruesse E., Yilmaz P., Gerken J., Schweer T., Yarza P., Peplies J., Glöckner F.O. (2013). The SILVA ribosomal RNA gene database project: Improved data processing and web-based tools. Nucleic Acids. Res..

[26] Ondov B.D., Bergman N.H., Phillippy A.M. (2011). Interactive metagenomic visualization in a Web browser. BMC Bioinform..

[27] Oliveira R.R.D.S., Fermiano D., Feres M., Figueiredo L.C., Teles F.R.F., Soares G.M.S., Faveri M. (2016). Levels of candidate periodontal pathogens in subgingival biofilm. J. Dent. Res..

[28] Socransky S.S., Haffajee A.D., Cugini M.A., Smith C., Kent R.L. (1998). Microbial complexes in subgingival plaque. J. Clin. Periodontol..

[29] Darveau R.P. (2010). Periodontitis: A polymicrobial disruption of hosthomeostasis. Nat. Rev. Microbiol..

[30] Kumar P.S., Griffen A.L., Moeschberger M.L., Leys E.J. (2005). Identification of candidate periodontal pathogens and beneficial species by quantitative 16S clonal analysis. J. Clin. Microbiol..

[31] Shi B., Chang M., Martin J., Mitreva M., Lux R., Klokkevold P., Sodergren E., Weinstock G.M., Haake S.K., Li H. (2015). Dynamic changes in the subgingival microbiome and their potential for diagnosis and prognosis of periodontitis. mBio.

[32] Shi M., Wei Y., Hu W., Nie Y., Wu X., Lu R. (2018). The subgingival microbiome of periodontal pockets with different probing depths in chronic and aggressive periodontitis: A pilot study. Front. Cell. Infect. Microbiol..

[33] Griffen A.L., Beall C.J., Campbell J.H., Firestone N.D., Kumar P.S., Yang Z.K. (2012). Distinct and complex bacterial profiles in human periodontitis and health revealed by 16S pyrosequencing. ISME J..

[34] Abusleme L., Dupuy A.K., Dutzan N., Silva N., Burleson J.A., Strausbaugh L.D., Gamonal J., Diaz P.I. (2013). The subgingival microbiome in health and periodontitis and its relationship with community biomass and inflammation. ISME J..

[35] Camelo-Castillo A.J., Mira A., Pico A., Nibali L., Henderson B., Donos N., Tomás I. (2015). Subgingival microbiota in health compared to periodontitis and the influence of smoking. Front. Microbiol..

[36] Liu B., Faller L.L., Klitgord N., Mazumdar V., Ghodsi M., Sommer D.D., Gibbons T.R., Treangen T.J., Chang Y., Li S. (2012). Deep sequencing of theoral microbiome reveals signatures of periodontal disease. PLoS ONE.

[37] Wang J., Qi J., Zhao H., He S., Zhang Y., Wei S., Zhao F. (2013). Metagenomic sequencing reveals microbiota and its functional potential associated with periodontal disease. Sci. Rep..

[38] Baker G.C., Smith J.J., Cowan D.A.G. (2003). Review and re-analysis of domain-specific 16S primers. J. Microbiol. Methods.

[39] Chakravorty S., Helb D., Burday M., Connell N., Alland D. (2007). A detailed analysis of 16S ribosomal RNA gene segments for the diagnosis of pathogenic bacteria. J. Microbiol. Methods.

[40] Ong S.H., Kukkillaya V.U., Wilm A., Lay C., Ho E.X., Low L., Hibberd M.L., Nagarajan N. (2013). Species identification and profiling of complex microbial communities using shotgun Illumina sequencing of 16S rRNA amplicon sequences. PLoS ONE.

[41] Pei A.Y., Oberdorf W.E., Nossa C.W., Agarwal A., Chokshi P., Gerz E.A., Jin Z., Lee P., Yang L., Poles M. (2010). Diversity of 16S rRNA Genes within Individual Prokaryotic Genomes. Appl. Environ. Microbiol..

[42] Sanz-Martin I., Doolittle-Hall J., Teles R.P., Patel M., Belibasakis G.N., Hämmerle C.H.F., Jung R.E., Teles F.R.F. (2017). Exploring the microbiome of healthy and diseased peri- implant sites using Illumina sequencing. J. Clin. Periodontol..

