# Supplementary material for: The Subgingival Microbiome in Patients with Down Syndrome and Periodontitis
Source: J Clin Med. 2020 Aug 2;9(8):2482. doi: 10.3390/jcm9082482 (PMC7463899; doi:10.3390/jcm9082482)
Supplement: Supplementary file 1 [file jcm-09-02482-s001.zip › suppl/Figure S1.pdf]

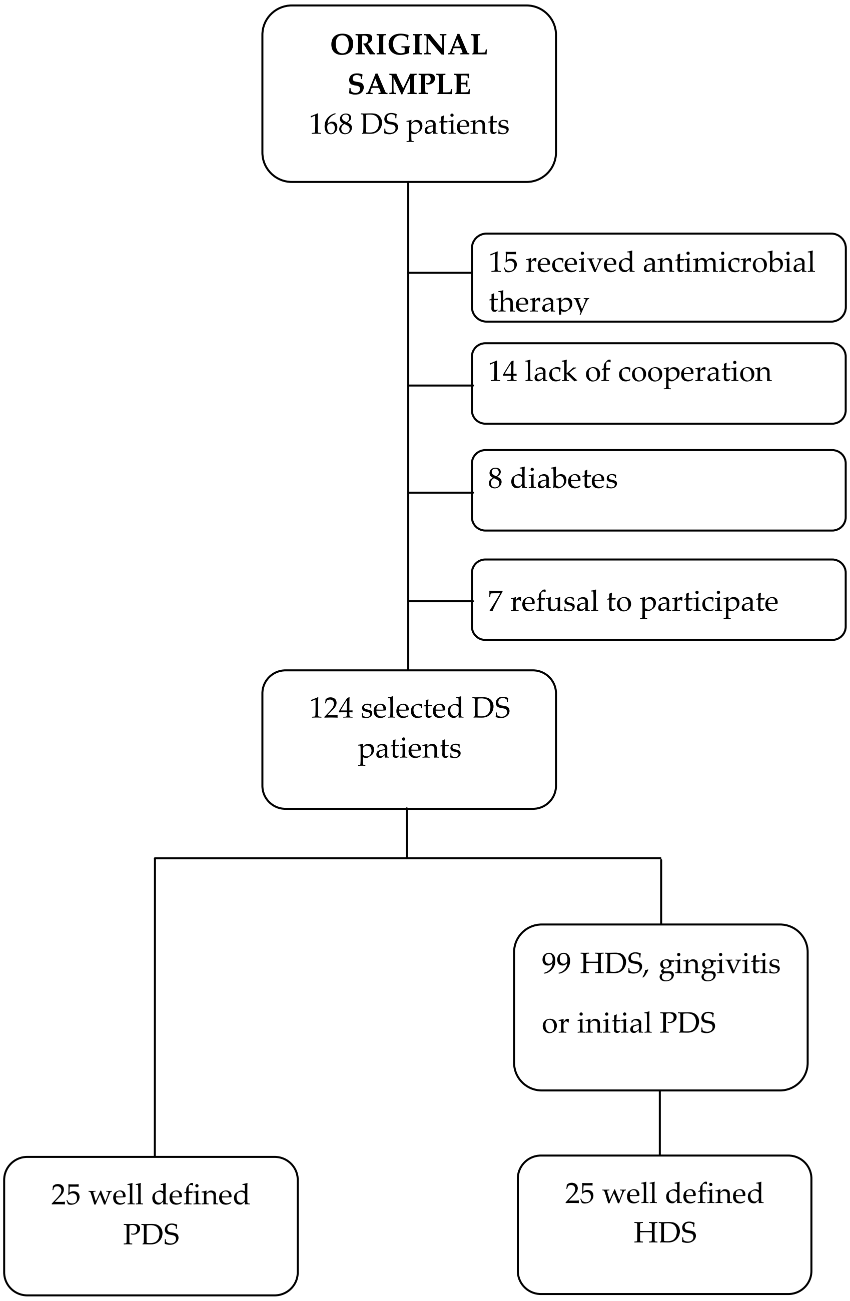

Figure S1. Flow diagram for the selection of individuals with Down syndrome included in this study with periodontitis (PDS) and healthy periodontium (HDS).
