# Supplementary material for: The Subgingival Microbiome in Patients with Down Syndrome and Periodontitis
Source: J Clin Med. 2020 Aug 2;9(8):2482. doi: 10.3390/jcm9082482 (PMC7463899; doi:10.3390/jcm9082482)
Supplement: Supplementary file 1 [file jcm-09-02482-s001.zip › suppl/Table S1.docx]

**Table S1.** Differences in microbial relative abundance (in percentages) evaluated using the Wilcoxon and Kruskal-Wallis tests between samples from patients with Down syndrome and

periodontitis or a healthy periodontal condition at the phylum, genus and species levels. Only taxa that were significantly different between the two groups (p≤0.05) are presented.

| **Taxa** | **Proportion in HDS** | **Proportion in PDS** | **Wilcoxon.pr** | **Kw.pr** |
| --- | --- | --- | --- | --- |
| **Phylum** | | | | |
| *Proteobacteria*  *Synergistetes*  *Chloroflexi* | 25.3127  0.1145  0.0017 | 13.3584  0.8059  0.0731 | 0.0416*  0.0381*  0.0119* | 0.0407*  0.0371*  0.0114* |
| **Genus** | | | | |
| *Atopobium*  *Olsenella*  *Cryptobacterium*  *Slackia*  *Phocaeicola*  *Paludibacteraceae F0058*  *Rikenellaceae RC9 gut group*  *Tannerella*  *Flexilinea*  *Lactococcus*  *Defluviitaleaceae UCG-011*  *[Eubacterium] nodatum group*  *Peptoanaerobacter*  *Selenomonas*  *Selenomonas 4*  *Gracilibacteria bacterium oral taxon 871*  *Saccharimonadaceae* spp.  *Lautropia*  *Ralstonia*  *Pseudomonas*  *Fretibacterium* | 0.0352  0.0263  0  0.002  0.0057  0.5967  0.0009  0.7171  0.0017  0.0021  0.0158  0.0757  0.0305  0.8424  0.1253  0.0037  0.05  0.4438  0.3784  4.7763  0.1145 | 0.165  0.0871  0.0036  0.0129  0.0845  2.2401  0.0802  1.6662  0.0731  0.0005  0.1127  0.2434  0.1998  1.3658  0.267  0  0.2995  0.1284  0.2128  0.029  0.7993 | 0.0289*  0.0085**  0.0412*  0.0203*  0.0172*  0.0004***  0.0119*  0.0298*  0.0119*  0.0498*  0.0434*  0.0361*  0.0307*  0.0397*  0.0198*  0.0209*  0.0034**  0.0055**  0.0344*  0.0029** | 0.0282*  0.0082**  0.0392*  0.0197*  0.0166*  0.0004***  0.0114*  0.029*  0.0114*  0.048*  0.042*  0.0352*  0.0299*  0.0388*  0.0193*  0.0198*  0.0033**  0.0053**  0.0336*  0.0028**  0.0371* |
| **Species** | | | | |
| *Actinomyces graevenitzii F0530*  *Actinomycetaceae F0332 unidentified*  spp.  *Atopobium unidentified*  spp.  *Olsenella unidentified*  spp.  *Olsenella sp. oral taxon 807*  *Cryptobacterium unidentified*  spp.  *Slackia sp. S4-C6*  *Phocaeicola abscessus CCUG 55929*  *Paludibacteraceae F0058 uncultured bacterium*  *Porphyromonas* spp*. oral clone CW034*  *Alloprevotella uncultured Prevotellaceae bacterium*  *Prevotella denticola*  *Prevotella 7 uncultured organism*  *Prevotella sp. oral taxon G60*  *Uncultured Prevotellaceae bacterium*  *Bacteroidales oral clone MCE7_164*  *Tannerella forsythia*  *Chloroflexi bacterium oral taxon 439 isolate Chl2*  *Lactococcus unidentified*  spp.  *Streptococcus anginosus subsp. anginosus*  *Defluviitaleaceae UCG-011 unidentified*  spp.  *Eubacterium minutum*  *Eubacterium nodatum*  *Mogibacterium timidum*  *Uncultured bacterium*  *Peptococcus sp. oral clone MCE10_265*  *Eubacterium sp. oral clone BB142*  *Selenomonas unidentified*  spp.  *Leptotrichia sp. oral clone IK040*  *Gracilibacteria bacterium oral taxon 871*  *Saccharimonadaceae unidentified*  spp.  *Lautropia uncultured bacterium*  *Ralstoniapickettii*  *Neisseria unidentified*  spp.  *Pseudomonas unidentified*  spp.  *Treponema 2 unidentified*  spp.  *Treponema sp. canine oral taxon 356*  *Treponema sp. oral taxon 265*  *Uncultured Deferribacteraceae bacterium* | 0.0074  0.0114  0.0352  0.0004  0.0259  0  0.002  0.0057  0.5967  0.0942  0.0179  0.2018  0.0027  0.0528  0.168  0.0003  0.1458  0.0017  0.0021  0.2635  0.0158  0  0.0464  0.0021  0.0509  0.1337  0.2994  0.2994  0.0771  0.0037  0.05  0.4108  0.3784  2.3348  4.7763  0.0659  0  0  0.0366 | 0.0022  0.0785  0.165  0.013  0.0741  0.0036  0.0129  0.0845  2.2401  0.1653  0.0411  0.3795  0.0104  0.1058  0.2071  0.0321  1.1464  0.0731  0.0005  0.4381  0.1127  0.0059  0.1607  0.0065  0.1193  0.2377  0.6188  0.6188  0.3935  0  0.2995  0.1161  0.2128  0.8287  0.029  0.3156  0.0221  0.0627  0.1479 | 0.0286*  0.0373*  0.0289*  0.0023**  0.0471*  0.0412*  0.0203*  0.0172*  0.0004***  0.0469*  0.0147*  0.0495*  0.0441*  0.0057**  0.0394*  0.0396*  0.0023**  0.0119*  0.0498*  0.0241*  0.0434*  0.0412*  0.0134*  0.0286*  0.0466*  0.0279*  0.0263*  0.0263*  0.0379*  0.0209*  0.0034**  0.027*  0.0344*  0.0378*  0.0029**  0.0235*  0.0412*  0.0209*  0.0326* | 0.0275*  0.0363*  0.0282*  0.0022**  0.046*  0.0392*  0.0197*  0.0166*  0.0004***  0.0458*  0.0143*  0.0483*  0.0426*  0.0055**  0.0385*  0.0381*  0.0022**  0.0114*  0.048*  0.0235*  0.042*  0.0392*  0.0129*  0.0275*  0.0455*  0.0272*  0.0256*  0.0256*  0.0369*  0.0198*  0.0033**  0.0263*  0.0336*  0.0369*  0.0028**  0.0229*  0.0392*  0.0198*  0.0316* |

Abbreviations: HSD, Down syndrome individuals with healthy periodontium; PSD, Down syndrome individuals with periodontitis.

Significant differences: *< 0.05, **<0.01, ***<0.001
