# Supplementary figures and images for: The Subgingival Microbiome in Patients with Down Syndrome and Periodontitis
Source: J Clin Med. 2020 Aug 2;9(8):2482. doi: 10.3390/jcm9082482 (PMC7463899; doi:10.3390/jcm9082482)

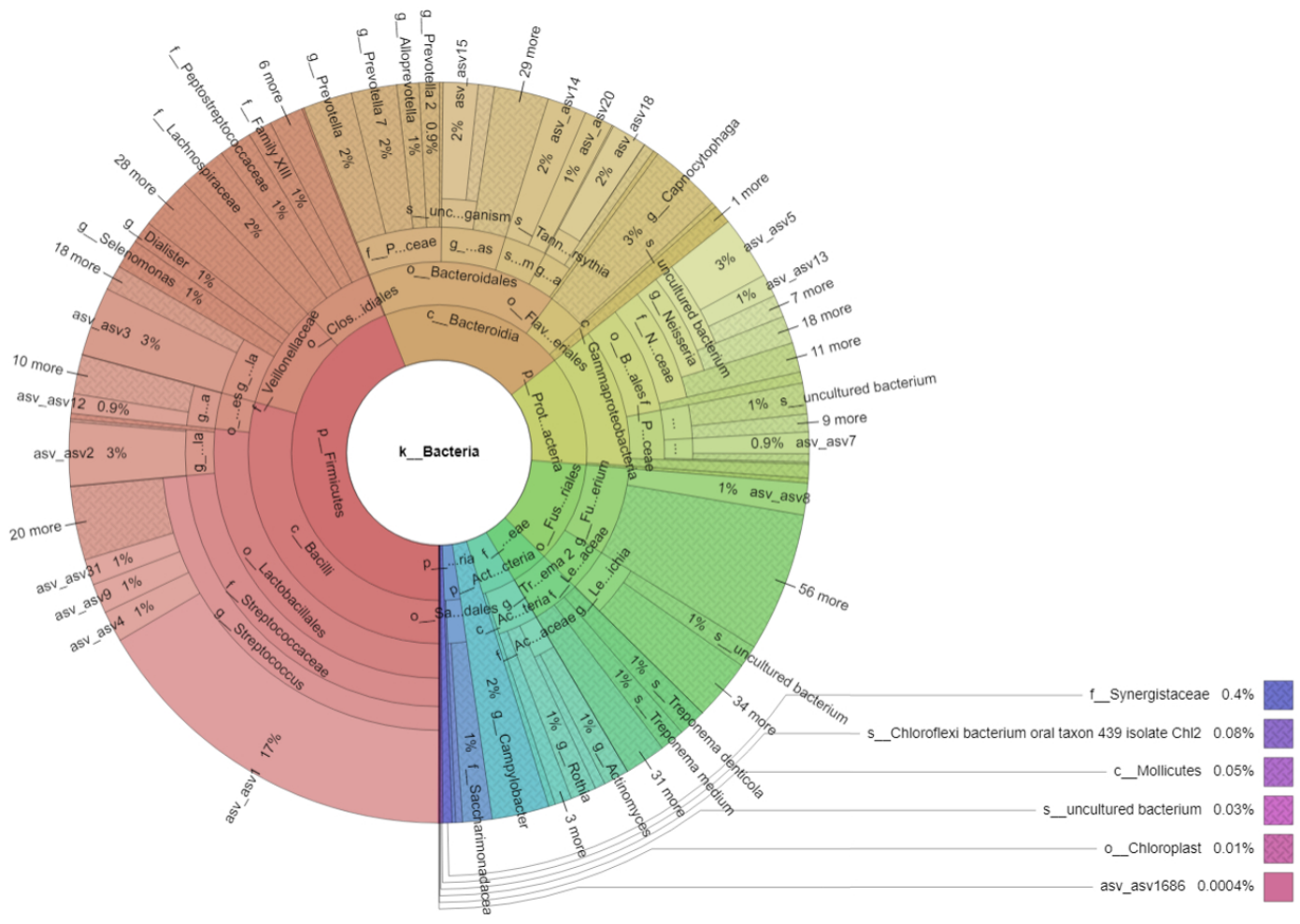

Supplement: Supplementary file 1 [file jcm-09-02482-s001.zip › suppl/Figure S2(A).pdf]

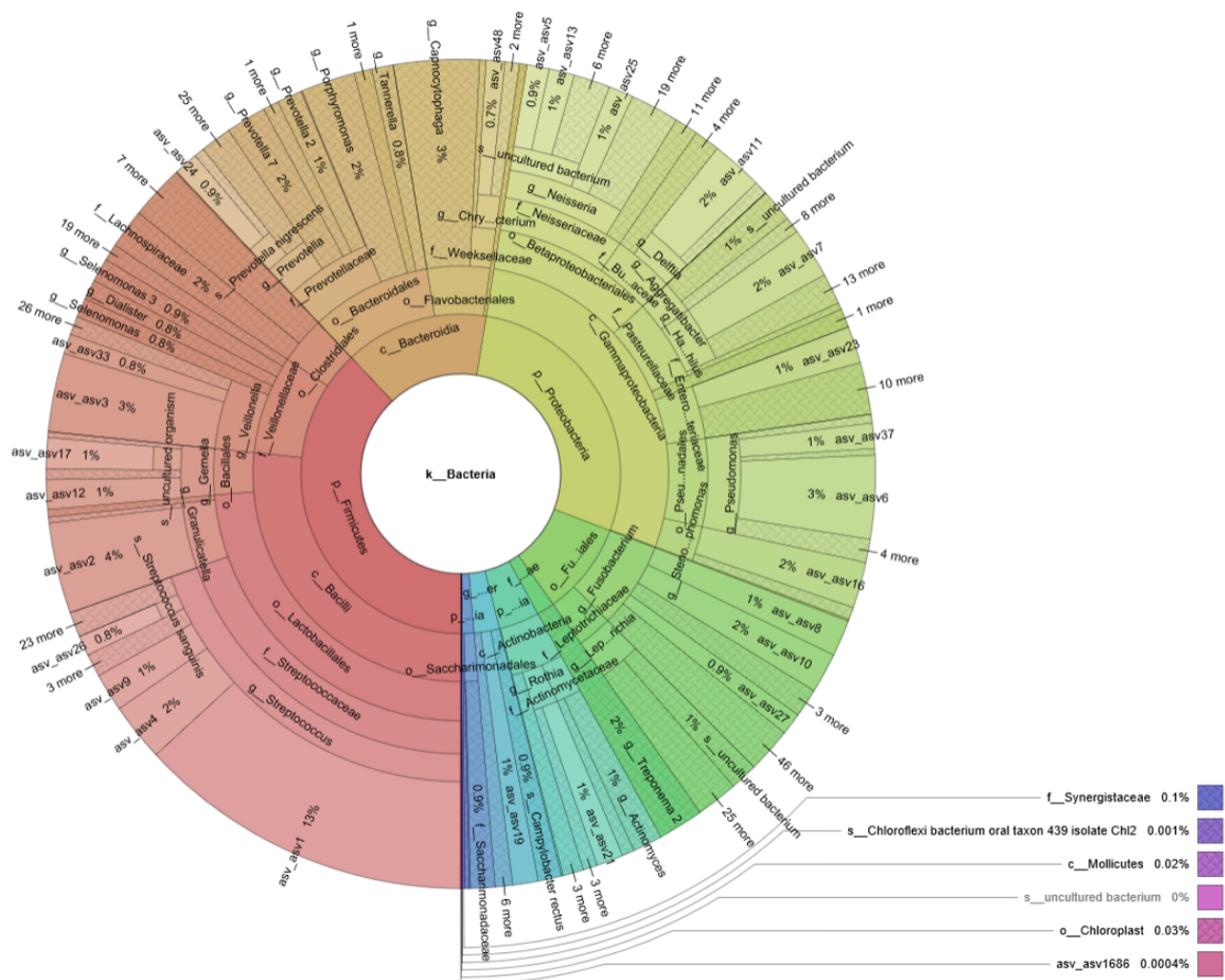

Supplement: Supplementary file 1 [file jcm-09-02482-s001.zip › suppl/Figure S2(B).pdf]

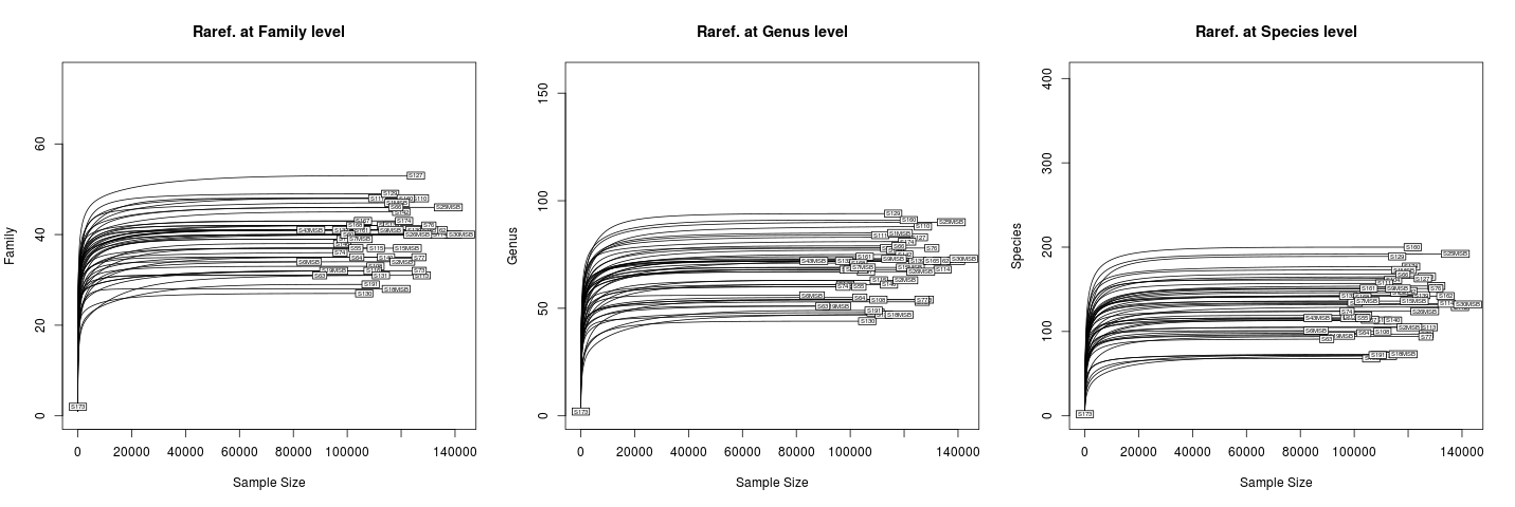

Supplement: Supplementary file 1 [file jcm-09-02482-s001.zip › suppl/Figure S3(A).tif]

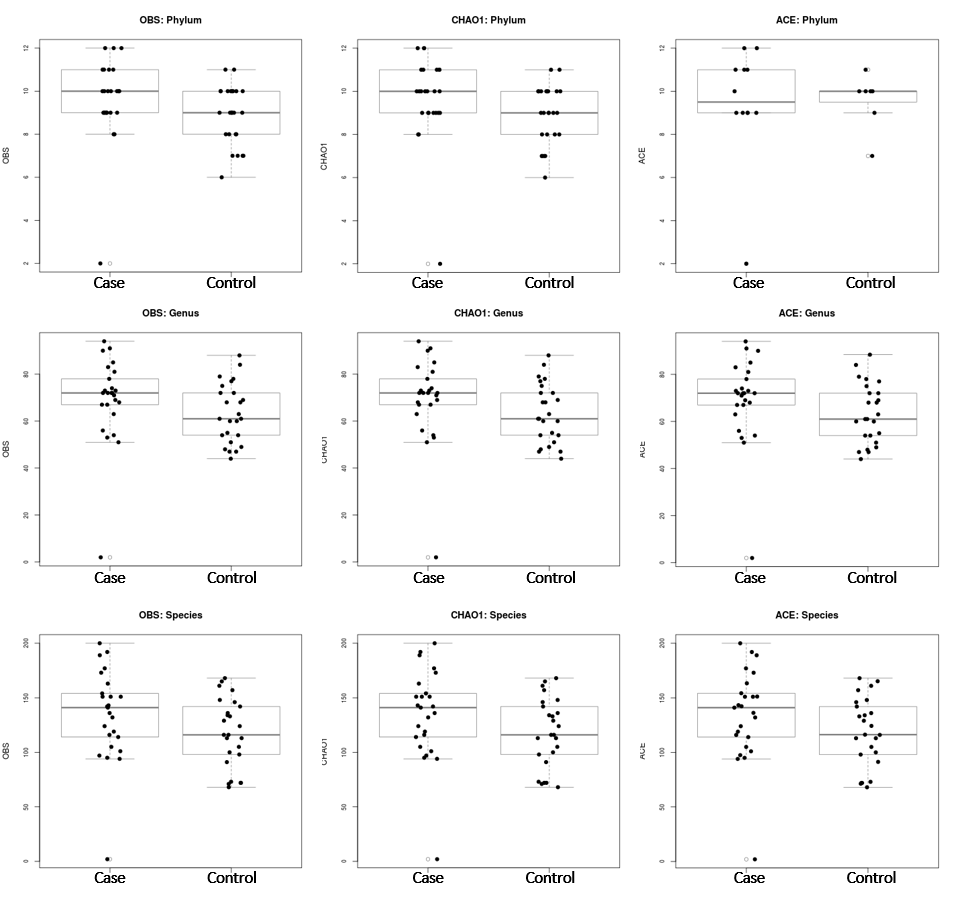

Supplement: Supplementary file 1 [file jcm-09-02482-s001.zip › suppl/Figure S3(B).tif]

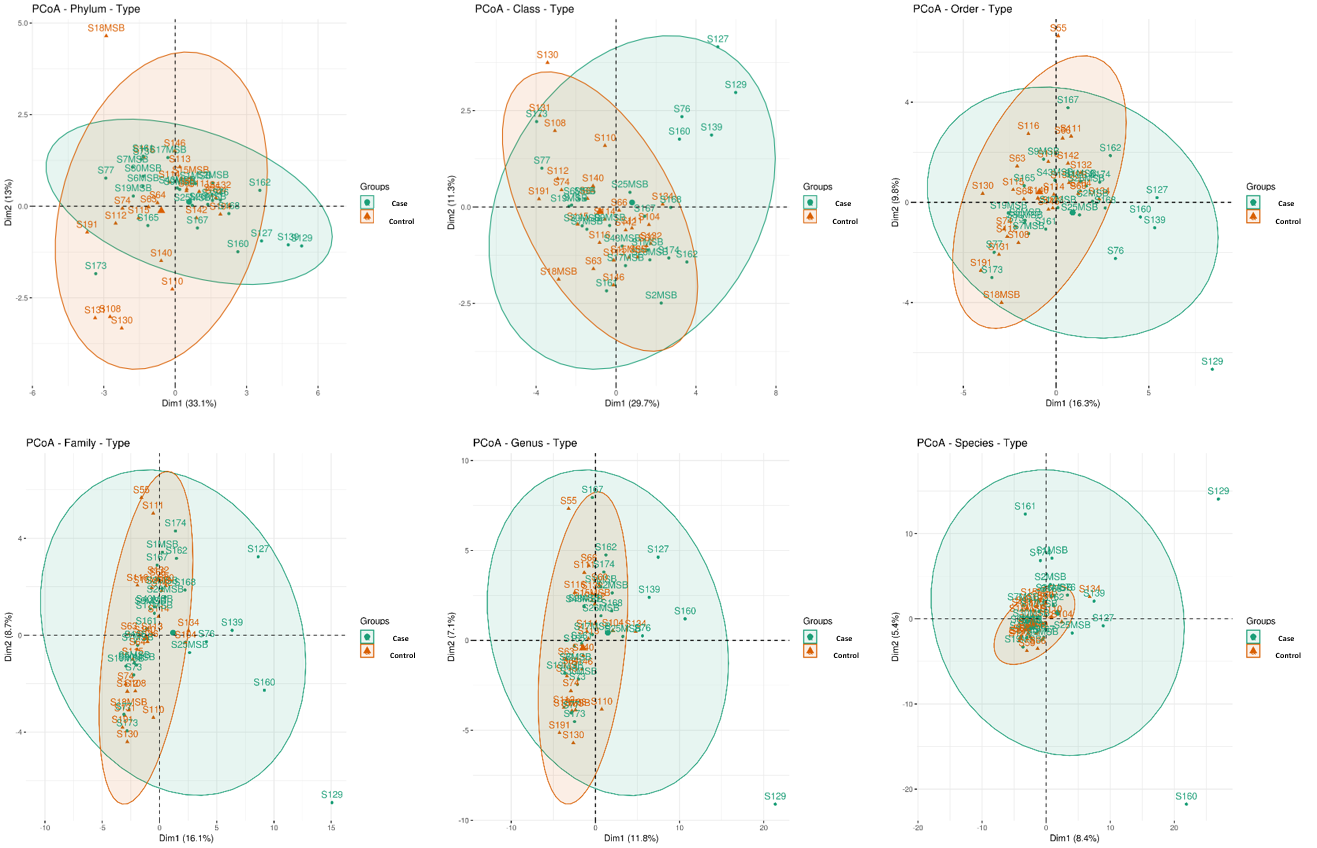

Supplement: Supplementary file 1 [file jcm-09-02482-s001.zip › suppl/Figure S4.png]
